# A preceptorship model to facilitate clinical nursing education in health training institutions in Botswana

**DOI:** 10.4102/curationis.v44i1.2182

**Published:** 2021-03-23

**Authors:** Antonia Dube, Mahlasela A. Rakhudu

**Affiliations:** 1 Department of Nursing, Faculty of Health Sciences, North-West University, Mafikeng, South Africa; 2 Department of Nursing, Faculty of Nursing, Kanye Seventh Day Adventist College of Nursing, Kanye, Botswana

**Keywords:** clinical nursing education, health training, facilitate, model, preceptorship, support

## Abstract

**Background:**

Despite the wide use of preceptorship, there is evidence that preceptorship and the role of preceptor in clinical nursing education are not clearly understood or supported.

**Objectives:**

To develop a preceptorship model to facilitate clinical nursing education in Botswana.

**Method:**

The model development in this study followed the steps of theory generation as described by Chinn and Kramer. These four steps are concept analysis, relationship statements, description and critical reflection of the model.

**Results:**

Four main themes emerged from the empirical study that formed the basis for key concepts and model development. The model has six components, namely, agent, recipient, context, procedure, dynamics and terminus. The description of the model is based on Chinn and Kramer.

**Conclusion:**

The need for a preceptorship model to facilitate preceptorship cannot be overemphasised in this regard. This model will guide the planning and implementation of preceptorship procedures by different stakeholders to improve its effectiveness in clinical nursing education.

## Introduction

Preceptorship is a leading clinical approach and support model that is currently used in undergraduate nursing education globally to facilitate the clinical learning process (Newton et al. 2012:2331). Despite the wide use of preceptorship, its use and the role of preceptor in clinical nursing education are neither clearly understood nor supported (Staykova, Husto & Pennington 2013:33). Myrick et al. (2011:134) contend that ‘the preceptorship approach to teaching-learning in the clinical environment provides an excellent modality in cultivating practical wisdom and ultimately to shape the art of nursing’. This supports the importance of using relevant preceptorship models to facilitate preceptorship. Cosme and Valente (2013:601) argued that although the preceptor plays a key role in the socialisation of the student in clinical nursing education, preceptors receive no or inadequate training or preparation. Furthermore, there is evidence suggesting that the role takes time to develop and is stressful with limited resources and support (Hallin & Danielson 2009:161; Horton et al. 2012:E2; Hyrkas, Liscott & Rhudy 2014:131). The cited studies accentuate the importance of preceptor preparation which is often embraced in contextual preceptorship models.

Kristofferzon et al. (2013:1251), affirm that support structures that include nurse facilitators, clinical lecturers and evaluated models to facilitate clinical education are of paramount importance to the students’ learning process. Kristofferzon et al.’s (2013) study was limited to students and did not include preceptors and nurse educators. These findings concur with other studies (Bourgeois, Drayton & Brown 2011:116; Gidman et al. 2011:351). The current study seeks to bridge the gap by including preceptors and nurse educators as key role players.

Consistent with other studies, Sen Gupta et al. (2008:17) assert that preceptor training established working guidelines for preceptors, disclosure of students’ limitations to preceptors, faculty support, training of preceptors on clinical teaching and assessment skills, and access of preceptors to information sources are key to the success of preceptorship. The aforesaid factors can be achieved through the use of well-structured models that facilitate preceptorship implementation in the context of the setting where clinical learning activities take place.

Mothiba et al. (2012:199–203) cited the following challenges associated with the implementation of preceptorship in South Africa that are consistent with the preceding international studies; unavailability of preceptors, large numbers of students, nurses’ lack of interest to work with students, poor interpersonal relations between students, staff work overload, inappropriate communication and students not accorded learning opportunities but rather assigned routine ward or clinic duties. Findings are in line with Horton et al. (2012:E5) that preceptors had to ‘carry a full load of patients and being a preceptor, whilst charge nurses and other staff members did not fully appreciate this added responsibility’.

In Botswana, all the six (6) health training institutions (HTIs) utilise preceptorship as a clinical teaching strategy for higher diploma in general nursing programme for level 3 students during clinical internship. The preceptor role is considered a mandatory responsibility for every registered nurse with a minimum of 2 years of working experience. However, there is no evidence of an existing model to support the preceptor role and preceptorship. This view is consistent with the preceptor selection criterion for nursing and midwifery in the United Kingdom which requires the nurse to have been on the Nursing and Midwifery Council (NMC) Register for Nurses, Midwives and Health Visitors for a minimum of 1 year (Panzavecchia & Pearce 2014:1119). Nurses in Botswana are often appointed by their supervisors to become preceptors upon request by the HTIs. The appointment is usually done regardless of whether the nurses have shown interest in the preceptorship role and often with minimal preparation for the role and multiple other nursing work-related responsibilities (Dube & Jooste 2006:130). This process compromises the quality of clinical nursing education especially if the appointed preceptor lacks interest in the role, skill in clinical teaching and supervision of students. Despite the aforesaid challenges and the inception of preceptorship over two decades ago, there has been little improvement on preceptorship modalities. There is still no preceptorship model to guide and improve preceptorship implementation in Botswana.

Contrary to the above study, respondents in Madisa (2012:124) cited three preceptor selection criteria whereby 5% of the preceptors was selected by students, 10% by managers and 30% by HTI whilst 55% volunteered to take up the role. These findings indicate a non-structured system with inadequate guiding principles that might have negative impact on the students’ clinical learning process. This necessitated the need for model development to strengthen preceptorship in clinical nursing education.

## Statement of the problem

Prior to the introduction of the alternative nursing education system in Botswana, the nurse educators were responsible for both classroom teaching and clinical follow-up of students in the clinical settings. This teaching modality put a strain on the nurse educators and compromised the quality of student’s clinical learning and supervision. Because of increased enrollment of students in HTIs, the need for preceptorship became evident and inevitable. In 1990, pre-registration nursing programme evaluation by the Ministry of Health in consultation with the Kellogg Foundation recommended preceptorship as a clinical teaching approach which was introduced in 1994. The large numbers of students enrolled increased the faculty–student ratio (Botswana 1995a).

Despite the existence of preceptorship in Botswana for over two decades, there is paucity of literature on preceptorship in Botswana. The few studies (Lwatula 2011; Madisa 2012:13; Monareng, Jooste & Dube 2009:114) highlight the lack of resources, inadequate preparation for the role, lack of clear policies and guidelines for preceptorship, lack of interest in the preceptor role, lack of support for preceptorship from the supervisors and colleagues of the preceptors, increased preceptor workload, lack of incentive or rewards for preceptors, inadequate follow-up by nurse educators to guide preceptors and students and unclear objectives of preceptorship as major setbacks. The lack of a preceptorship model and guidelines is the key factor contributing to the setbacks associated with the implementation of preceptorship.

### Purpose

The purpose of this study was to develop the preceptorship model for facilitating clinical education in HTIs in Botswana.

### Significance of the study

The findings from this study have a potential to inform the best nursing education practices in Botswana and guide the restructuring of preceptorship in clinical nursing education for the pre-service and post-basic students. The findings may inform the formulation of clear preceptorship policies. Recommendations from the study have a potential for further research on identified areas to close the existing gaps in preceptorship in clinical nursing education.

## Method and materials

### Study Setting

The study was conducted in selected clinical settings which were utilised as clinical teaching sites for level 3 higher diploma in general nursing students during preceptorship and all six HTIs in Botswana. Preceptorship activities for students occur under the supervision of nurse preceptors in the clinical settings. Nurse educators from HTIs act as resource personnel to facilitate for and support students and preceptors during preceptorship.

### Research methods

The current study used a mixed method and focused on the preceptors as key supervisors of nursing students and the nurse educators who provide pedagogical support to students and preceptors. The preceptors and nurse educators described and explained their experiences during preceptorship. Their findings were used to develop a preceptorship model to facilitate clinical nursing education for Botswana.

The model development in this study followed the four-step theory generation as described by Chinn and Kramer (2011:163–182). These four steps are concept analysis, relationship statements, description and the critical reflection of the model.

#### Step 1: Concept analysis

**FIGURE 1:**
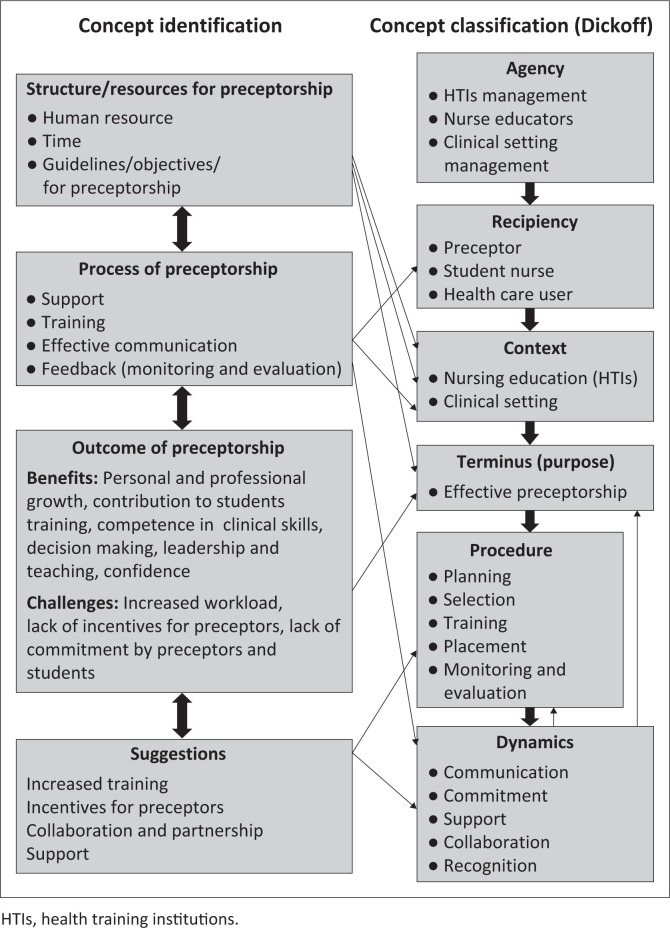
Classification of the concepts according to Dickoff’s model.

The concept analysis was conducted in two stages, namely, central concept identification through empirical process and classification of related concepts. This step followed fieldwork in empirical study using a convergent mixed method and literature review. The identified concepts were classified according to Dickoff, James and Wiedenbach’s (1968:422–423) elements for theory development. Figure 1 depicts the classification of the concepts from the empirical study according to Dickoff et al. (1968) survey list.

#### Step 2: Relationship statement

The identified concepts in step 1 were put into interrelated statements (Chinn & Kramer 2012:180). Figure 1 displays the relationships. Concepts and relationships between concepts provided the foundation for developing the model.

#### Step 3: Model description

**FIGURE 2:**
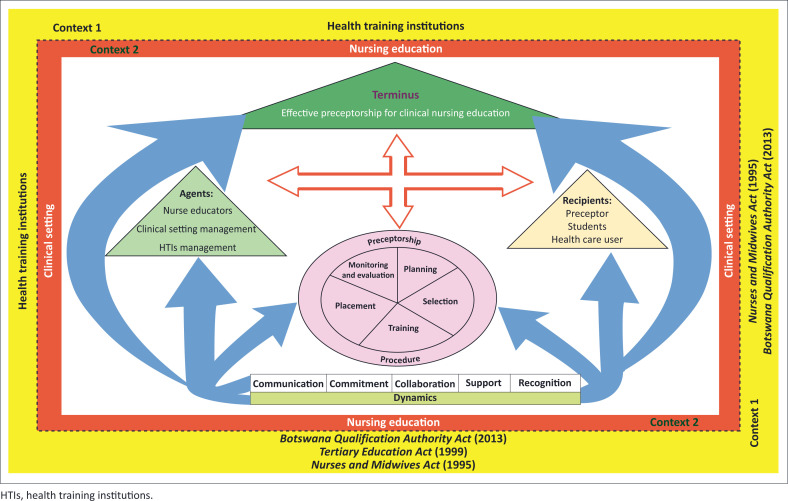
A precetorship model to facilitate clinical nursing education in Botswana.

The model is based on Chinn and Kramer’s (2012:184–196) components of a theory, namely, purpose, concepts definition, relationship statements, nature of the structure and assumptions. The model is applicable in the clinical context of nursing education facilitated through implementation of preceptorship in clinical education. Figure 2 shows the preceptorship model to facilitate clinical nursing education in Botswana.

#### Step 4: Critical reflection

Chinn and Kramer (2012:196–204) advocate for critical reflection to ascertain how well a model works in relation to the intended purpose and contributes to understanding how well the framework relates to educational activities, research and practice. The evaluation criteria addressed the clarity, simplicity, accessibility generality and importance of the model (Chinn & Kramer 2012:185–192)

### Quality measures

Content validity was checked with nursing education experts from North-West University and Botswana Kanye College of Nursing. The questionnaire was piloted with 10 participants and adjusted accordingly. In the qualitative component member checking, the use of a coder and engaging a qualitative research expert and audit trails were employed to ensure trustworthiness. Preliminary findings were shared with participants for their feedback to validate the findings and the final conclusions were made. An audit trail was maintained by keeping the filed notes, voice recorded tapes, coded data, themes that emerged from the codes and interpretations drawn from the agreed upon discussion.

### Ethical considerations

Ethical approval was granted by the North-West University Ethics Committee (NWU 00250-15-A9) and Health Research Unit, Ministry of Health in Botswana (NO: HPDME 13/18/1). Organisational authorities of the clinical settings and Health Training Institutions acted as gatekeepers. Informed consent was obtained by mediators identified by gate keepers and participation was voluntary.

## Results and discussions

The results are given according to the steps in the model development.

### Step 1: Concept analysis

Four main themes and 12 sub-themes emerged from the empirical phase, namely, structure, process and outcomes of preceptorship as well as suggestions to improve the preceptorship in clinical education. The themes were corroborated in the quantitative components of the mixed method. These empirical results and literature review formed the basis for concept classification and model development. The central concept from data analysis is preceptorship, and the related concepts included structural, process and outcome issues. Table 1 depicts the merged results which formed the basis for the model development.

**TABLE 1:** Preceptors’ and nurse educators’ perceptions on preceptorship.

Theme	Sub-theme	Quantitative results
Inadequate resources for preceptorship	Inadequate human resource Increased time pressure No clear guidelines for preceptors	The majority of respondents (91.0%) agreed that they would recommend a model of preceptorship to facilitate clinical nursing education in Botswana. Furthermore, 84.0% agreed that preceptorship needs much restructuring for it to meet its intended objective.
Inadequate processes to facilitate preceptorship	Lack of support for preceptors Unstructured preceptorship Ineffective communication	Eighty per cent of nurse educators show that preceptors’ co-workers and supervisors are not supportive of the goals of preceptorship. Fifty seven per cent of preceptors and 86% of nurse educators indicated minimal support of preceptors from nurse educators.
		Nurse educators indicated that not all preceptors are trained or oriented for the role whilst 78.0% disagreed that preceptors were confident to evaluate students’ clinical activities objectively.
		A total of 56.6% (*n* = 87) respondents disagreed that there is clear and easy communication between nurse educators and preceptors. One important finding was that 68.0% of preceptors and only 49.5% of nurses’ educators disagreed that nurse educators and preceptors meet regularly to discuss issues pertaining to preceptorship.
Perceived outcomes of preceptorship	Benefits of preceptorship	87.8% of respondents revealed that being a preceptors keeps the preceptor up to date and stimulated in the professional role.
		87.9% preceptors showed that their analytic and critical thinking skills greatly improved by being preceptors.
		85.1% of preceptors indicated that being a preceptor improved their teaching skills.
	Challenges to effective preceptorship	The majority (61.7%) preceptors indicated that they do not have sufficient time to provide patient care whilst functioning as a preceptor and that preceptors’ other responsibilities do not allow them to attend to students’ learning needs.
Suggestions for improvement of preceptorship	Empowerment and training of preceptors	Almost all (98%) of participants suggested continuous training and development of preceptor as well as collaborative partnerships with structure such as regulatory authorities.
	Collaboration with key stakeholders Recognition for preceptors	Eighty seven per cent (87%) indicated the need for recognition of preceptors and provision of incentives for their work.

Findings are supported by preceptorship literature globally (Mårtensson et al. 2013:446; Mothiba et al. 2012:199; Tan et al. 2011:19) wherein lack of preceptors and increased workload were highlighted as concerns that can impact negatively on the learning environment.

Findings are congruent with Panzavecchia and Pearce (2014:1121–1122) whereby 83% of respondents indicated that time was a major barrier to effective preceptorship and should be considered in planning the working time of the preceptor and preceptee against the workload of the preceptor.

Horton et al. (2012:E5) emphasised the importance of support of preceptors to enable them to effectively execute the preceptorship role as a cornerstone of clinical nursing education and students’ supervision. Likewise, Hallin and Danielson (2009:161–162) highlighted that the success of preceptors in their preceptorship role depends to a greater extent on the support they get, their interest in the role, preceptors’ experiences in the role and the preceptorship model used.

The findings from both preceptors and nurse educators support literature evidence that many preceptors are not adequately prepared for their role, particularly in the areas of teaching and evaluating students (Horton et al. 2012:E2; Hovland 2011:3; Hyrkas et al. 2014:120; Kristofferzon et al. 2013:1253; Mårtensson et al. 2013:448; Panzavecchia & Pearce 2014:1120–1121).

Lack of communication verbalised by participants in this study is incongruent with the findings and recommendations from other studies (Iglesias-Parra et al. 2015:4; Madisa 2012:49; Mothiba et al. 2012:202; O’Brien 2015:18). The importance of establishing frequent communication between clinical preceptors and faculty or educational institutions as a way to facilitate success for preceptorship is mandatory.

In support of preceptors’ view of the benefits of preceptorship, nurse educators agreed with the following; ‘Preceptorship provides professional growth and development for preceptors’; (76%); ‘preceptorship has more benefits for students clinical learning than the traditional faculty-student model of clinical teaching’ (68%). To further justify the benefits of preceptorship, nurse educators (78%) disagreed that ‘there are no benefits associated with preceptorship as a clinical teaching strategy’. The findings support literature that preceptorship benefits for the preceptor include keeping up to date as they learn new technology from students and stimulating thinking, satisfaction with the professional role, personal and professional growth (Billay & Myrick 2008:258; Connor 2015:337; Shepard 2014:73).

In support of the incentives for preceptors, Rose (2008:106–107) and Tan et al. (2011:18) highlighted that recognition can be offered as a luncheon, certificates, invitations to recognition events, or even a letter as a token of appreciation for the job well performed. Findings also corroborate with Liu et al. (2010:807) who posited that provision of ongoing professional development activities may be a valuable strategy to replace the monetary rewards.

#### Concept classification

The main concepts from study results are identified and classified according to Dickoff et al. (1968:422–423). Six elements of practice theory are the agent, recipient, context, procedure, terminus and dynamics and their related question. Figure 1 shows the classification of concepts for a preceptorship model developed to facilitate clinical nursing education.

### Step 2: Relationships statement

The identified and defined concepts in step 1 were put into interrelated statements (Chinn & Kramer 2008:246–248, 2012:180). The relationship statements formulated for this preceptorship model to facilitate clinical education are given below:

- Preceptorship in clinical education requires addressing of the structural issues such as human resource, time and guidelines/ objectives/ for preceptorship within the context (HTIs and clinical setting).
- Critical to preceptorship are the process standards such as support, training, effective communication, feedback of monitoring and evaluation by the agents and recipient of preceptorship. Agents in preceptorship include HTI management, nurse educators and clinical setting management whilst the recipients include preceptors, nursing students and healthcare users.
- The outcomes of preceptorship are positive, but challenges are also inevitable. Positive outcomes include following benefits (1) personal and professional growth; (2) contribution to students training, (3) competence in clinical skills, decision-making, leadership and teaching and (4) increased self-confidence.
- Challenges in preceptorship include (1) increased workload, (2) lack of incentives for preceptors and (3) lack of commitment by preceptors and students.
- For achievement of the benefits of preceptorship, processes such as planning, appropriate selection, training and placement of preceptors as well monitoring and evaluation of the whole preceptorship process are critical.
- Successful preceptorship is dependent upon the following: collaboration between key role players, namely; agents and recipients, support for preceptor and preceptorship from managers of clinical settings and HTIs, effective communication between preceptors and nurse educator and students.

### Step 3: Model description

The preceptorship model is described in terms of purpose, overview, structural description, nature and assumptions (Chinn & Kramer 2012:184–196).

#### Purpose of the clinical model

Consideration of who will implement it and under what conditions or circumstance (Chinn & Kramer 2012:185) is important. It is envisaged that this preceptorship model will be used as a framework of reference to facilitate clinical teaching and guide HTIs and clinical settings on the guidelines for operationalisation of the model.

#### Structure of the model

Figure 2 is a schematic representation of the preceptorship model to facilitate clinical education, including the agents who should guide the preceptorship, the beneficiaries of the process that could be followed to achieve the preceptorship, dynamics or factors that could help to drive the preceptorship process, the context necessary to support it and the outcomes that should be expected from the preceptorship.

#### The nature of the preceptorship model

The preceptorship model is multidimensional and consists of six elements of Dickoff et al. (1968) as depicted in Figure 2.

The symbolic meanings of the diagram are as follows:

- The outer frame around the model represents the context wherein preceptorship will occur, namely, HTIs, and inner frame represents nursing education in the clinical settings where preceptorship activities are offered.
- The arrows symbolise the relationships or influence on each element of the model, namely, context, agents, recipients, process, dynamics and terminus.
- The circular structure represents the preceptorship process or procedure which is a continuous process. Included are planning of preceptorship, selection, placement, and training of preceptors, as well as monitoring and evaluation of the preceptorship process.
- The smaller triangles represent the key role players in preceptorship, namely, agents (HTI management, nurse educators and clinical managers) and recipients (preceptors, nursing students and healthcare users).
- The bigger triangle represents the terminus or goal of the model, which is effective preceptorship for clinical nursing education.
- The four directional arrows between the terminus, recipient, agents and process indicate the associational relationships amongst the stated elements within the context of preceptorship, namely, agents, recipients, process and terminus.
- The multiple arrows from the dynamics to various elements of the model indicate the underlying powers that influence the elements positively (agents, recipients, process and terminus) within the context of HTIs, nursing education and clinical settings.
- The dynamics facilitate effective preceptorship process, namely, planning, selection, training and placement of preceptors and monitoring and evaluation of preceptorship. Dynamics are the factors that drive all the elements or components of the preceptorship to facilitate its effectiveness.

#### Assumptions of the model

Assumptions are related to the relationship statement and reflect the values underlying the model. The following are the assumptions upon which the model is based:

**Assumptions related to context:** Preceptorship activities occur in two contexts, namely, HTIs where students are enrolled for training as nurses and the clinical settings where preceptors work and supervise students during clinical learning.

The outer box represents nursing education in the HTIs, whilst the inner box represents the clinical settings context. The perforated line between the HTI and clinical context indicates the close relationship between two contexts. Nursing education context is regulated by legal instruments, namely, the *Botswana Qualification Authority Act* No. 24 (2013), the *Nurses and Midwives Act* (1995b) and the *Tertiary Education Act* (1999).

**Assumptions related to agent and recipients:** Agents determine who is to perform the activity. In this preceptorship model, the agents shall refer to nurse educators, management of clinical settings and management of HTIs.

In the implementation of this preceptorship model to facilitate clinical nursing education, recipients are students and preceptors and indirect receipts are healthcare users. Students and preceptors are primary recipients of preceptorship practices.

The structure of preceptorship should be such that the relationship between the learning context, HTIs and clinical setting management, nurse educator and the preceptor should facilitate learning opportunities for the student during preceptorship.

The preceptor should provide adequate orientation to students on clinical setting policies that regulate the practice of nursing. To enable them to implement such policies during preceptorship, the student shall implement and comply with all academic and professional policies and regulations that govern their clinical nursing education practice.

**Assumptions related to procedure:** In this model, procedure refers to the preceptorship processes and all activities to be performed to reach the terminus or purpose of the preceptorship model which is to facilitate clinical nursing education. In the context of this model, the procedure for preceptorship includes activities such as planning, selection of preceptors, training; placement of preceptors and monitoring and evaluation of preceptorship activities.

**Assumptions related to dynamics:** Dynamics of preceptorship in the model include communication, support collaboration, commitment and recognition. These dynamics that exist during preceptorship within the context of HTIs or clinical setting positively influence how the agent and recipients interact for effectiveness of preceptorship.

Suggestions for improvement of preceptorship include support from managers of HTIs and clinical settings, collaboration between key stakeholders, namely, nurse educators, preceptors, clinical settings and HTIs management. Effective communication between nurse educators and preceptors and recognition of the preceptorship role through incentives for preceptors was also highlighted as crucial to effective preceptorship.

Communication, support, collaboration, commitment between the agents and recipients are forces that drive preceptorship to facilitate clinical education.

### Step 4: Critical reflection of the model

The model was not evaluated by experts. However, a critical reflection was done using Chinn and Kramer’s (2012:204) five (5) critical reflection elements.

#### Clarity

The semantic clarity of the model was achieved through the use of only core concepts of preceptorship; no new concepts were introduced (Chinn & Kramer 2012:204). Structural clarity has been achieved by the use of Dickoff et al.’s (1968:422–423) activities as the basis for describing the structure of the model.

#### Simplicity

This model is relatively simple to understand as the structural components and their relationships are clearly indicated and explained (Chinn & Kramer 2012:205). The procedure of how the model will facilitate preceptorship including the dynamic forces that exist within the contexts of nursing education and clinical settings and their influence on the effectiveness of preceptorship in clinical nursing education is clearly stated in simple terms.

#### Generality

It refers to its breath of scope, purpose, its applicability and broad array of situations (Chinn & Kramer 2012:202). Although the purpose of the preceptorship model is to facilitate clinical nursing education, it can also be used in other nursing contexts such as practice and administration particularly in the implementation of new initiatives and programmes. The model can also be used in any learning and teaching situations that requires students to be supervised by qualified staff in the real work situation.

#### Accessibility

It is the extent to which the indicators for concepts can be identified and to what extent the purpose can be attained (Chinn & Kramer 2012:203). The model will be presented to nurse educators, management of HITs and clinical settings in workshops, conferences and seminars for them to appreciate the model and its purpose.

#### Importance

The importance of the model is linked to its significance or practical value (Chinn & Kramer 2012:202). The model aims to improve the effectiveness of preceptorship by addressing identified challenges in the education and clinical contexts such as inadequate resources, lack of expertise in role performance, lack of support, lack of commitment and ineffective communication.

## Practical implications

Although the model was developed in the context of preceptorship in clinical nursing education in Botswana which is a developing country with limited resources, the model can also be used in other countries within the Southern African Development Community (SADC) where clinical nursing education still relies on the utilisation of nurses in the clinical setting to supervise students. The model can also be used in other developing countries within Africa and globally encountering similar challenges in the implementation of preceptorship.

## Recommendations

Recommendations are made in relation to nursing education, nursing practice, nursing research and policymaking.

### Nursing education

Preceptorship is the core clinical teaching strategy used in Botswana and in many other counties regionally and globally for senior nursing students. The restructuring of preceptorship to embrace the learning needs of students and teaching needs of preceptors should be guided by a preceptorship models developed based on each country’s context.

Regular and effective communication between nurse educators and preceptors to discuss preceptorship and students is recommended as a way of motivation and helping preceptors and students improve on identified weaknesses.

### Nursing practice

The clinical environment should provide enabling and supportive procedures for the recipients to facilitate effective preceptorship. It is, therefore, recommended that:

- Collaborative partnerships between clinical settings and HTIs must be strengthened for effective support to preceptors.
- Formal and clear guidelines for selection of preceptors should be developed in line with the desired characteristics of a preceptor.
- Nursing education standards as prescribed by regulatory bodies should be implemented.

### Nursing research

The recommendations for research include:

- Implementation and critical reflection of the preceptorship model to establish relevance and effectiveness.
- Developing an evaluation tool to test the model within the Botswana’s clinical nursing education context.

### Policymaking

The policymakers in the health sector are the custodians of nursing education and practice and have an obligation to ensure best practices, quality of nursing education and practice in the country. Recommendations for policymaking are:

- The model could be used to guide selection and placement of preceptors within the clinical settings.
- Incentives to be included as a package for preceptorship to attract nurses to take up the role of preceptor.
- Development of job descriptions for preceptors for role clarity.

## Limitations of the study

The study was conducted only in the context of Botswana and may require adjustments for other regions if replicated because of differences in context. However, important lessons can still be learned from the study.

The convenience sampling technique employed for the quantitative component limits the generalisability of findings.

## Conclusion

Nursing is a practice-based discipline with clinical practice being central to nursing education. A conclusion could be reached that the preceptorship model developed in this study will greatly contribute to facilitating clinical nursing education and effectiveness of preceptorship. Training and support for preceptors and nurse educators should be promoted for effective preceptorship. Giving preceptors’ incentives may motivate more nurses to develop interest in taking up the preceptorship role and alleviate the current problem of unavailability of preceptors. The rigour and intensity of the study were ensured and the results are gratifying in their unique contribution to the body of knowledge specifically to nursing education, nursing practice, policymaking and the nursing profession as a whole. Because of new trends in nursing and nursing practice, both the nursing education and clinical setting environments are continuously evolving, making them more complex and challenging. The model seeks to provide an alternative decision to address the challenge entailed thereof.
